# Anti-NMDA Receptor Encephalitis following Herpes Simplex Encephalitis presenting as Mania with Psychotic Features: Case Report

**DOI:** 10.1192/j.eurpsy.2025.733

**Published:** 2025-08-26

**Authors:** I. M. Lopes, D. Seabra, G. Santos, N. Ramalho, T. Coelho Rocha, J. Alves Leal, J. F. Cunha, J. Moura, D. Santos, A. Garcia, M. Rosa, C. Pires

**Affiliations:** 1 Psychiatry, ULS Arco Ribeirinho; 2 Psychiatry, USL Arco Ribeirinho; 3 Psychiatry, ULS Barreiro, Barreiro, Portugal

## Abstract

**Introduction:**

Herpes Simplex Encephalitis (HSE) and anti-NMDA receptor autoimmune encephalitis (ANMDARE) are severe neurological conditions that can lead to significant psychiatric symptoms. While these conditions commonly cause cognitive and behavioral disturbances, mania with psychotic symptoms is an uncommon manifestation. Understanding this rare presentation is crucial for accurate diagnosis and management.

**Objectives:**

To describe a case of mania with psychotic symptoms in a 33-year-old woman approximately one month after Herpes Simplex Encephalitis (HSE), further complicated by anti-NMDA receptor autoimmune encephalitis.

**Methods:**

We conducted a detailed review of the clinical process and heteroanamnesis from family reports. A non-systematic literature review was performed using the terms “encephalitis,” “mania,” “psychosis,” “herpes simplex,” and “anti-NMDA” in the PubMed®/MEDLINE® database.

**Results:**

A 33-year-old woman, seven months postpartum with no prior psychiatric history, presented with psychomotor agitation, distractibility, elevated mood, verbose speech, tachypsychia, impulsivity, verbal perseveration, insomnia, and mystical and persecutory delusions, including auditory-verbal hallucinations, starting 15 days after discharge from hospitalization for HSE. Her subsequent hospitalization revealed severe and fluctuating behavioral changes, significant memory deficits, and spatial-temporal disorientation. Neuroimaging showed atrophy of the left temporal lobe, ipsilateral insula, and notable involvement of the left hippocampus. Cerebrospinal fluid analysis detected anti-NMDA receptor antibodies, leading to treatment with corticosteroids and immunoglobulins. The patient was stabilized on clozapine 150 mg/day, valproate 1000 mg/day, clonazepam 1 mg/day, and monthly injectable risperidone 100 mg.

**Conclusions: Discussion:**

HSE is a major cause of death in sporadic encephalitis cases, with a 12% relapse rate linked to viral reactivation and the development of anti-NMDA receptor encephalitis. ANMDARE, caused by anti-NMDA receptor IgG antibodies targeting the NR1 subunit, affects about 25% of HSE patients within three months. Psychiatric affective syndromes have been described both as possible initial symptoms of HSE and as long-term sequelae, but the underlying mechanisms remain not fully understood.

**Disclosure of Interest:**

None Declared

